# Frailty before and during austerity: A time series analysis of the English Longitudinal Study of Ageing 2002–2018

**DOI:** 10.1371/journal.pone.0296014

**Published:** 2024-02-07

**Authors:** Carys Pugh, Chima Eke, Sohan Seth, Bruce Guthrie, Alan Marshall

**Affiliations:** 1 Advanced Care Research Centre, Usher Institute, University of Edinburgh, Edinburgh, United Kingdom; 2 School of Social and Political Science, University of Edinburgh, Edinburgh, United Kingdom; Instituto Nacional de Geriatria, MEXICO

## Abstract

**Background:**

Frailty is characterised by a reduced resilience to adversity. In this analysis we examined changes in frailty in people aged 50+ before and during a period of austere public spending in England.

**Methods:**

Data from the English Longitudinal Study of Ageing 2002–2018 were analysed. Associations between austerity and frailty were examined using (1) Multilevel interrupted times series analysis (ITSA); and (2) Accelerated longitudinal modelling comparing frailty trajectories in people of the same age in 2002 and 2012.

**Results:**

The analysis included 16,410 people (mean age 67 years, 55% women), with mean frailty index score of 0.16. Mean scores in women (0.16) where higher than in men (mean 0.14), and higher in the poorest tertile (mean 0.20) than the richest (mean 0.12). In the ITSA, frailty index scores increased more quickly during austerity than before, with the additional increase in frailty 2012–2018 being similar in magnitude to the difference in mean frailty score between people aged 65–69 and 70–74 years. Steeper increases in frailty after 2012 were experienced across the wealth–spectrum and in both sexes but were greater in the very oldest (80+). In the accelerated longitudinal analysis, frailty was lower in 2012 than 2002, but increased more rapidly in the 2012 cohort compared to the 2002 cohort; markedly so in people aged 80+.

**Conclusion:**

The period of austerity politics was associated with steeper increases in frailty with age compared to the pre–austerity period, consistent with previously observed increases in mortality.

## Introduction

In 2008/9, a financial crisis in the US spread quickly across the world, forcing governments in many countries to borrow significant sums of money to prevent collapse in the banking sector and broader economy. Subsequently, policies of austerity were implemented to varying degrees in many countries as governments sought to reduce budget deficits through combinations of increased taxes and/or reduced public spending. In the UK, the government focused particularly on reducing public spending, making cuts across government spending from 2010 including welfare [1], local government budgets [2], and social care [3]. NHS spending increased from less than 5% of GDP in the early 2000s to a highpoint of 7.6% in 2009–10 but austerity was a factor here too and spending began to fall slightly as a percentage of GDP during the 2010s while the population continued to age [4]. Research has suggested that the impact of austerity, while most focused on the poorest people [5], also affected more affluent groups [1]. Beyond the obvious impacts such as waiting lists for elective surgery growing from 2.5 million people in April 2012 to 4.1 million people in 2017 [6], researchers have connected the broad and significant extent of austerity in the UK to a range of concerning trends including rising use of food banks [7], deterioration in community infrastructure, services and environment quality [5, 8], increasing barriers to receipt of social care services [9], deterioration in indicators of mental health and rising suicide, and elevated levels of homelessness and housing insecurity [10]. Cuts to local government spending have been directly associated with increasing levels of multimorbidity [11] and, perhaps the starkest potential impact of austerity, is the levelling off in the rate of increase in life expectancy from 2014 (with decline for some groups) [12], following 200 years of increasing life expectancy [13, 14].

Frailty is where an individual has a limited homeostatic reserve and is most commonly a consequence of multi–system decline over a lifetime [15]. Frailty is an important outcome in later life since it leads to a lack of resilience and reduced capacity to recover from shocks, meaning that relatively minor events can trigger major changes in health status. Frailty is associated with multiple adverse consequences including increased risk of falls [16], hospitalisation [17], increased length of hospital stay [18], institutionalisation [19], poorer quality of life [20], and death [21–23]. A body of literature has demonstrated increasing frailty with age, higher frailty among women compared to men, and stark and widening inequalities in frailty across indicators of socio–economic position (for example, Harttgen and colleagues [24] and Marshall and colleagues [25]). Research on changes in frailty over time have found little evidence for improvement in levels of frailty, despite general trends of improved longevity. For example, results comparing Swedish cohorts born 30 years apart found similar levels of frailty [26] and, more pessimistically, frailty was observed to be higher in later–born US [27, 28], Netherlands [29] and Hong Kong Chinese [30] population cohorts compared to earlier born. These findings are in line with a broader body of research across a wide range of health indicators that shows growing inequalities in health, with improvements in healthy life expectancy lagging improvements in life expectancy [31, 32]. The bulk of evidence appears in line with the *expansion of morbidity* theory, whereby life expectancy increases faster than healthy life expectancy [33].

Understanding change in frailty before and during austerity is important as it provides a marker of the health of the older population and their longer–term capacity for independent living. In the context of austerity and population ageing, there is little health and social care capacity to cope with increasing frailty. This paper uses nationally representative data from the English Longitudinal Study of Ageing (2002 to 2018), to examine whether frailty increased at different rates during austerity than before. Our work was written according to the guidelines developed by the STROBE initiative [34].

## Methods

We hypothesised that the introduction of austerity policies in 2010 would be associated with steeper increases in frailty with age. We used multilevel interrupted time series analyses to explore whether the change in frailty with age was different after the implementation of austerity policies compared to before, with a sensitivity analysis using an accelerated longitudinal design to compare changes in frailty for different birth cohorts, at the same age, from 2002 and 2012.

### Data source

The data were from the English Longitudinal Study of Ageing (ELSA) [35], a cohort study following individuals two–yearly from 2002. Data were collected by a mix of face-to–face interview and nurse assessments. Data collected for core ELSA members across nine waves from 2002–2019 were used, including individuals who joined via refreshment sampling in waves 3, 4, 6, 7 and 9. Each wave of data collection took place across two years, for example, wave nine was collected in 2018 and 2019, but specific data collection dates were not accessed for this analysis, so all data collected during a particular wave was treated as though it was collected in the first year of data collection (i.e. 2002, 2004… 2018). The authors did not have access to data that could identify participants at any stage.

### Frailty index score

The outcome was a frailty index score based on Rockwood’s [36] frailty deficit model approach, whereby the score is the proportion of deficits assessed which are present in the individual, ranging from 0 (no deficits) to 1. The frailty index score is considered a reasonable proxy for frailty with higher scores, like higher levels of frailty, associated with poor outcomes [37]. A score was ascertained for all participants, with individuals included for a specific wave of data collection provided data were available for more than 55 of 62 potential deficits (detailed in S1 Table). The distribution of frailty index scores was strongly right–skewed so, for modelling, values were square–root transformed.

### Other variables

Initial data exploration examined model fit using age as a continuous variable, both linear and polynomial. Linear age was a much poorer fit and the polynomial model was a no better fit than age fitted as a categorical variable which is easier to interpret. Participants were therefore grouped by age in 2002 (<55, 55–59, 60–64, 65–69, 70–74, 75–79, 80–84 and 85+). Participants were grouped into tertiles (three equally sized groups) of total net wealth excluding pension savings (ELSA variable nettotw_bu_s) with tertiles generated for each wave of data collection, and within groups according to the decade of birth (due to low numbers, those born before 1930 were considered together). The wealth variable included in the model therefore represents the relative wealth of the individual, at that time point, when compared to those of a similar age. As such, two people with identical net wealth in wave 1, might be in different wealth tertiles because they were born in different decades. Similarly, an individual’s wealth might not change between waves of data collection, but their wealth tertile may, because the relative wealth of others in their birth cohort changed. We also examined interaction terms, and the final model includes an interaction term for age*wealth tertile.

### Statistical analysis

For the primary interrupted time–series analysis, a linear multilevel model approach was used in R. The structure of our model had repeated measures at level 1 nested within individuals at level 2 (model detailed in S1 File). We fitted a random intercept model allowing the level of frailty to vary between individuals. A key advantage of using a mixed/multilevel model is that the approach is robust to unequal time spacing between observations, missing data, and has the flexibility for the inclusion of subject covariates that are either continuous or discrete measures and which are variant or invariant over time. We assessed how frailty index score (the proportion of deficits present) increased with age, how this varied by sex and wealth tertile, and whether the period characterised by austerity policies was associated with a change in the rate of increase in frailty index score over time as people aged.

The specification of the time-point for the intervention in the interrupted time–series is challenging because austerity policies occurred over a phase of time that differed across various areas of public spending, and because effects on frailty may take some time to develop. Beatty and Fothergill [1] identify a set of initial welfare reforms that occurred between 2010 and 2015, and we based our interruption on this, fitting models with the interruption in time series in 2010 (when austerity policies first began), 2012 and 2014 (by which time many austerity measures were embedded). Our results suggested that interruptions at all three time points give the same substantive conclusions, but model fit is best and our results strongest with an intervention in 2012 and we report these findings in the main paper (AIC values for 2010, 2012, 2014 given in S2 Table, model results in S3 and S4 Tables, and figures in S1 and S2 Figs for 2010 and 2014 respectively).

Three sensitivity analyses were completed. Firstly, we fitted an alternative accelerated longitudinal design (allowing us to compare the evolution of frailty for different birth cohorts, at the same age, from 2002 and 2012) to check that the findings of the interrupted time series modelling were not simply due to frailty changing non–linearly with age. The model is detailed in S2 File. Secondly, it might be argued that income, rather than wealth, is a better indicator of social circumstances, so modelling was repeated using income tertiles based on participant total income (totinc_bu_s) rather than wealth tertiles. The model results with income did not lead to any substantive differences in the model outcomes. Thirdly, frailty is a multidimensional measure and it is possible that changes in a small number of deficits or groups of deficits might drive results. We additionally examined changes in a frailty deficit score for each of the seven domains included in the overall frailty score (mobility, activities of daily life, cardiovascular disease, chronic illnesses, psychiatric illness, general wellbeing, and memory) (S1 Table).

### Ethical approval

Analysis used anonymised publicly available data under the conditions of ELSA ethics approval, and ethical review for this specific analysis was not required.

### Patient and public involvement

Two lay members were part of the advisory group who contributed to the design and conduct of the study. It is expected that they will also help frame the dissemination of the study findings.

## Results

**Fig 1:**
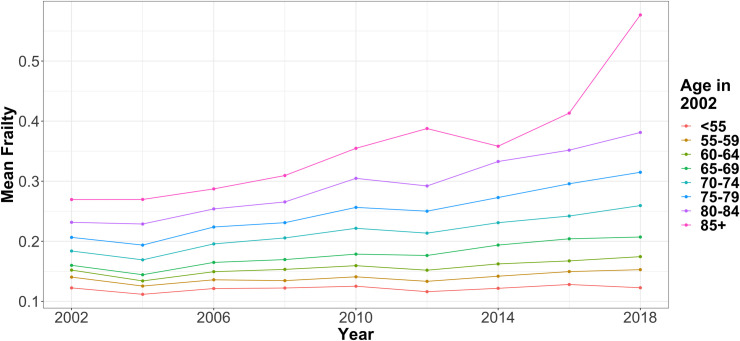
Mean frailty at each wave of data collection, stratified by age in 2002.

A total of 16,410 individuals were included in the analysis (8,977 women), with a mean age of 67 across all years (see Table 1). On average, each individual contributed 4.5 frailty index scores to the modelling (range 1–9, median 4). These scores ranged from 0–0.76 with a mean of 0.15 and a median of 0.12. Men had lower mean and median frailty index scores (0.14 and 0.11), compared to women (0.16 and 0.13). Raw mean frailty index scores over time are shown below (Fig 1), stratified by age in 2002 which is the strongest single determinant of frailty although with large variation between individuals.

**Table 1:** Characteristics of the population.

		Frailty score in 2002	Frailty score in 2012	Frailty score all time points
		Mean/Median/IQR (n)	Mean/Median/IQR (n)	Mean/Median/IQR (n)
Age	50–54	0.12/0.09/0.09 (1742)	0.11/0.07/0.09 (589)	0.12/0.08/0.09 (7176)
	55–59	0.14/0.11/0.11 (2121)	0.12/0.08/0.11 (1320)	0.13/0.09/0.10 (11885)
	60–64	0.15/0.11/0.12 (1617)	0.12/0.08/0.10 (1622)	0.13/0.10/0.10 (13194)
	65–69	0.16/0.12/0.12 (1647)	0.13/0.98/0.10 (1621)	0.14/0.11/0.11 (12759)
	70–74	0.18/0.15/0.13 (1416)	0.15/0.12/0.13 (1212)	0.16/0.13/0.12 (11305)
	75–79	0.20/0.18/0.14 (1060)	0.17/0.14/0.13 (1100)	0.18/0.15/0.14 (8563)
	80–84	0.23/0.20/0.16 (793)	0.20/0.18/0.17 (591)	0.21/0.19/0.16 (5750)
	85+	0.27/0.25/0.18 (374)	0.25/0.23/0.18 (457)	0.25/0.23/0.18 (3558)
	All ages	0.16/0.13/0.13 (10770)	0.14/0.11/0.13 (8515)	0.15/0.12/0.13 (74190)
Sex	Women	0.17/0.14/0.14 (5846)	0.15/0.09/0.11 (4744)	0.16/0.13/0.14 (41185)
	Men	0.15/0.12/0.12 (5846)	0.13/0.12/0.14 (3771)	0.14/0.11/0.11 (33005)
Wealth	Poorest	0.21/0.18/0.18 (3590)	0.19/0.16/0.18 (2823)	0.20/0.17/0.18 (24660)
	Middle	0.16/0.12/0.12 (3588)	0.13/0.10/0.12 (2851)	0.14/0.11/0.12 (24823)
	Richest	0.13/0.10/0.09 (3592)	0.11/0.08/0.09 (2841)	0.12/0.09/0.09 (24707)

### Interrupted time series model

**Fig 2:**
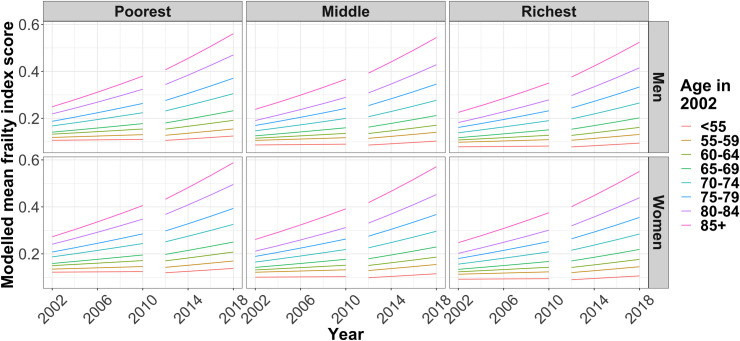
Modelled mean frailty over time for different age groups, stratified by wealth tertile and sex using an interrupted time series approach.

Model coefficients are in Table 2, but interpreting the individual coefficients is complicated by the square–root transformation, and the interaction terms. Fig 2 therefore shows the model predicted frailty index score (with model estimates squared to give mean frailty). On average, frailty index score increased strongly with age, was routinely higher for women than men of the same age, and was lower for richer people. The wealth effect was nonlinear with the poorest third having distinctly higher levels of frailty than the richest and middle thirds. For the youngest age groups, the difference was equivalent to the poorest third being as frail as people in the richest third of the population who were ten years older, but this association attenuated with age. The association between frailty index score and sex was weaker than that with wealth, with women having similar frailty in middle–age as men who were approximately five years older.

**Table 2:** Interrupted time series model output predicting the square root of the mean frailty index score.

*Predictors*	*Estimates (95% confidence interval)*	*P*
(Intercept)	0.280 (0.275 to 0.285)	**<0.001**
Sex (binary)	..	..
Men	Reference	..
Women	0.024 (0.020 to 0.028)	**<0.001**
Age in 2002 (years)	..	..
<55	Reference	..
55–59	0.028 (0.021 to 0.035)	**<0.001**
60–64	0.040 (0.033 to 0.048)	**<0.001**
65–69	0.053 (0.046 to 0.061)	**<0.001**
70–74	0.077 (0.068 to 0.086)	**<0.001**
75–79	0.096 (0.085 to 0.196)	**<0.001**
80–84	0.129 (0.116 to 0.141)	**<0.001**
≥85	0.162 (0.143 to 0.182)	**<0.001**
Wave (linear 1–9)	..	..
1 = 2002–03, 9 = 2018–19	0.002 (0.001 to 0.003)	**<0.001**
Wealth tertile (categorical)	..	..
Richest	Reference	..
Middle	0.014 (0.010 to 0.017)	**<0.001**
Poorest	0.044 (0.040 to 0.049)	**<0.001**
Period (binary 0,1)	..	..
0 = waves 1–5	Reference	..
1 = waves 6–9	-0.055 (-0.057 to -0.046)	**<0.001**
**Interactions**	..	..
Sex*Wave	..	..
Men*wave	Reference	..
Women*wave	-0.000 (-0.001 to -0.000)	**0.030**
Age in 2002*wave	..	..
50–54*wave	Reference	..
55–59*wave	0.002 (0.002 to 0.003)	**<0.001**
60–64*wave	0.006 (0.005 to 0.006)	**<0.001**
65–69*wave	0.009 (0.008 to 0.010)	**<0.001**
70–74*wave	0.012 (0.012 to 0.013)	**<0.001**
75–79*wave	0.016 (0.015 to 0.018)	**<0.001**
80–84*wave	0.019 (0.017 to 0.020)	**<0.001**
≥85*wave	0.025 (0.021 to 0.029)	**<0.001**
Wave * Period	0.007 (0.006 to 0.008)	**<0.001**
Age in 2002*Wealth	..	..
<55*richest	Reference	..
55–59*middle	-0.002 (-0.007 to 0.003)	0.467
60–64*middle	-0.001 (-0.006 to 0.005)	0.864
65–69*middle	-0.006 (-0.011 to 0.000)	0.064
70–74*middle	-0.003 (-0.010 to 0.004)	0.354
75–79*middle	0.001 (-0.008 to 0.009)	0.894
80–84*middle	-0.004 (-0.015 to 0.007)	0.433
≥85*middle	0.003 (-0.017 to 0.023)	0.792
55–59*poorest	-0.014 (-0.021 to -0.007)	**<0.001**
60–64*poorest	-0.011 (-0.019 to -0.003)	**0.004**
65–69*poorest	-0.014 (-0.022 to -0.006)	**<0.001**
70–74*poorest	-0.010 (-0.020 to 0.001)	**0.025**
75–79*poorest	-0.010 (-0.022 to 0.001)	0.068
80–84*poorest	-0.019 (-0.034 to 0.005)	**0.008**
≥85*poorest	-0.019 (-0.042 to 0.004)	0.109
**Random Effects**	..	..
σ^2^	0.004	..
τ_00__ij_	0.012	..
ICC	0.764	..
N_j_	16410	..
Observations	74190	..
Marginal R^2^ / Conditional R^2^	0.180 / 0.807	..

The rate of increase in frailty scores accelerated for all age groups following the best–fit modelled interruption of 2012 (see Fig 2). Over the period of four waves of data collection (2012–2018), the increase in frailty index score is approximately equivalent to an additional five years of ageing, similar in size to the difference between men and women. The estimated impact of austerity over the six years of follow–up is smaller than the estimated difference between the poorest and richest tertiles, but changes in frailty are seen across all wealth groups. As can be seen in Fig 2, the absolute increase was greater in the older age groups. For example, the mean frailty index score for the richest men aged 55–59 in 2002 was increasing by 0.0014 per year from 2002–2010, rising to 0.0038 per year from 2012–2018. By contrast, the mean frailty index score for the poorest women aged 80–84 in 2002 was increasing by 0.0098 per year from 2002–2010, rising to 0.015 per year from 2012–2018.

### Accelerated longitudinal modelling (ALM)

**Fig 3:**
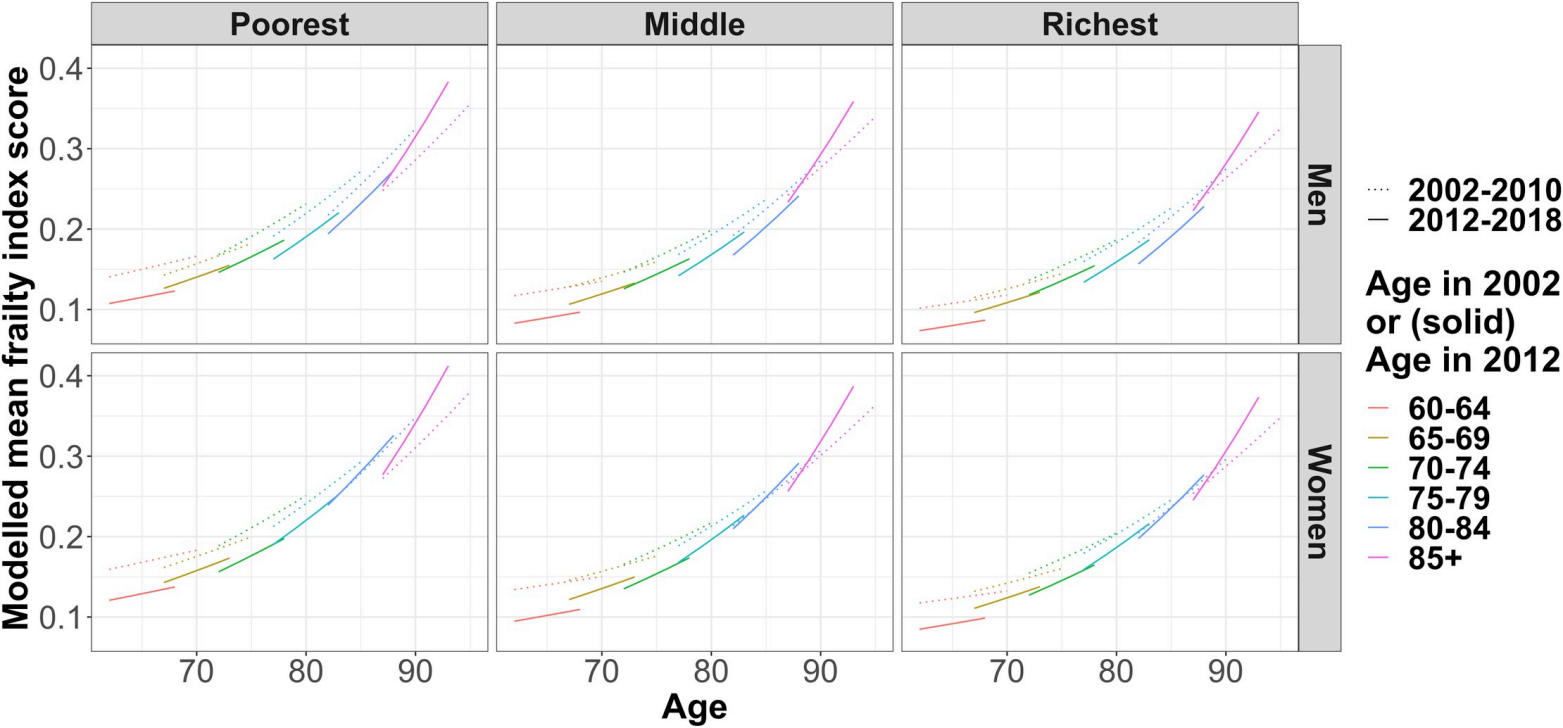
Modelled mean frailty over time for different age groups, stratified by wealth tertile and sex using an accelerated longitudinal design approach (2002–2010 and 2012-–2018 modelled separately).

Modelling the periods separately facilitates direct comparison of frailty trajectories between, for example, those who were 65–70 in 2002 and those who were 65–70 in 2012. The models are detailed in S1 File and their outputs are given in S5 Table and shown in Fig 3. Here people in 2012 start with lower frailty than their age-matched counterparts in 2002, suggesting that frailty had broadly improved during the 2000s, but frailty index scores then accrue more quickly for all age groups in the later time period. This is true for men and women, richer and poorer, but is particularly evident in the oldest ages groups. The increased rate of accrual results in average frailty scores in 2012–2018 going above the 2002–2010 levels for women aged 80+ and men aged 85+, meaning the oldest groups were frailer at the end of the 2010s compared to the equivalent age groups in the late 2000s.

### Other sensitivity analyses

Model results were not substantively different with income tertile as the predictor rather than wealth tertile and, whilst different domains changed at different ages, there was no evidence that changes in frailty post–austerity were driven by one or two specific domains (S3 Fig).

## Discussion

This study compared changes in frailty with age before and after implementation of austerity policies reducing public spending in many areas including welfare, social care and local government. The study finds increasing frailty with age, and higher frailty for women compared to men and for the poorest compared to the richest groups. In both the ITSA and ALM analyses, mean frailty score increases faster during the period of austerity (2012–2018) than in the pre–austerity period (2002–2010).

The key strength of this research is that we utilise a nationally representative social survey that includes a rich set of information from people born before the first world war to those born in the 1960s. We were able to construct a robust frailty index score and individual wealth measures, with sufficient data before and after austerity to estimate trends but there are several limitations to our research.

Firstly, the frailty index score is built using self-reported measures. Whilst much of the self-reporting comes from well-validated questionnaires, we cannot rule out changing trends in how people respond to such questions. Secondly, it might be argued that the increased rate of change in frailty from 2012 compared to before is simply a consequence of a non–linear increase in frailty as cohorts age. We explored this possibility by modelling trajectories before and during austerity separately, with age groups assigned by age in 2002 and 2012 respectively. Under this approach the 2012 model predicts faster accrual of deficits than the 2002 model, in line with the ITSA. Thirdly, ELSA is subject to non–random attrition as respondents choose not to take part in waves of the survey, they move into residential care, or they die. Those who drop out are more likely to be male, in poorer health and in lower socio–economic groups bringing potential bias to results. Since people with higher levels of frailty are more likely to drop out, we might expect our results to understate the extent of steepening in frailty trajectories after austerity compared to before, however it may also be the case that people have been supported to live in their own homes for longer, effectively increasing the frailty in the ELSA population but not the wider UK population. We were not able to control for this and it should not be dismissed as an issue, particularly in the oldest age groups.

Finally, as with all observational studies, this was an analysis of associations and causality cannot be strongly inferred. As was seen in previous work by Mousa and colleagues in England [38], changes in frailty may be driven by approaches to medical care that affect both ascertainment and prevalence of conditions. They compared frailty 1991 and 2011 and found higher levels of frailty in the 2011 group, driven by hypertension and diabetes, the former being better diagnosed and the later likely being both better diagnosed and increasing in prevalence. Whilst diabetes was not a key driver in our findings, we did not examine broader impacts of increases in sedentary behaviour and obesity which are also likely to be playing a role in changing health over time. We did however see frailty index score increase in all wealth groups (the richest typically having low levels of obesity) and our findings are consistent with other analyses finding associations between austerity and food insecurity [7], housing precarity [10], multi-morbidity [11] and mortality [12].

The UK experienced the highest (pre-pandemic) annual spike in mortality in 2015, which occurred predominantly among frail older people and was sustained in subsequent years [12]. Darlington–Pollock and colleagues [14] estimate that the levelling off in mortality rates from 2010–2018 cost around 230,000 lives compared to a continuation of prior trends, and increasing frailty is a logical precursor to these increased deaths. Mortality attributed to austerity in older groups occurred in all geographic areas (affluent and deprived) [12]. This has been echoed by our finding that frailty index score increases faster in all population groups (rich and poor), which accords with previous studies showing that austerity impacted across all population groups [1], although there is variation in the impact of austerity on excess mortality. For example, the extent of local cuts to social care and to financial support aimed at older people are associated with area level rises in mortality among those aged over 85 [12], which is the age group where we observe the largest change in frailty in the accelerated longitudinal design models.

A policy implication of our research is to recognise that public spending reductions have impacts on health and mortality across all population groups. Most governments internationally face challenging fiscal circumstances as societies emerge from the acute stages of the pandemic compounded by rising energy costs and general inflation. Proposals to cut public funding should therefore balance perceived benefits with likely consequences for health and other social outcomes. This study also finds that from the early 2000s to the early 2010s, when health spending was increasing, mean population frailty was falling, but this improvement was largely lost due to accelerated accrual of frailty deficits during austerity. Population resilience to further austerity in the 2020s may therefore be lower than it was in the 2010s.

There is a need for further research to better understand what caused the deterioration in health outcomes, such as frailty, during austerity compared to before. Although we did not find any one domain of frailty deficits to be driving results, future research might focus on specific deficits that one might expected to be particularly linked to austerity in the short term. Additionally, research exploiting the harmonised longitudinal data on older cohorts including the English Longitudinal Study of Ageing, the Health and Retirement Study in the USA, and the Survey of Health and Ageing and Retirement in Europe has the potential to explore differences in frailty trajectories in national contexts where different policy responses to the financial crisis were implemented. This has the potential to help evidence whether austerity policies were causally related to changes in frailty, as countries each approached the financial crisis differently. Such differences may also identify elements of 2010s austerity that were potentially the most harmful, facilitating mitigation should further austerity be pursued. If the political response to the pandemic and subsequent economic instability is further austerity, then further longitudinal research to examine its effects across a range of outcomes including frailty will be needed.

## Supporting information

S1 Table Elements comprising the frailty index score and their seven domains. (DOCX)

S2 Table AIC values for the different model interruption points in the interrupted time series analysis. (DOCX)

S3 Table Interrupted time series model output predicting the square root of the mean frailty index score. Interruption point is 2010.
(DOCX)

S4 Table Interrupted time series model output predicting the square root of the mean frailty index score. Interruption point is 2014.
(DOCX)

S5 Table Accelerated time series model outputs predicting the square root of the mean frailty index score. Modelled for 2002–2010 and 2012–2018 separately.
(DOCX)

S1 File Interrupted time series analysis model specification. (DOCX)

S2 File Accelerated times series model specification. (DOCX)

S1 Fig Modelled mean frailty over time for different age groups, stratified by wealth tertile and sex using an interrupted time series approach with interruption in 2010. (DOCX)

S2 Fig Modelled mean frailty over time for different age groups, stratified by wealth tertile and sex using an interrupted time series approach with interruption in 2014. (DOCX)

S3 Fig Mean proportion of deficits for each domain, at each wave of data collection, stratified by age in 2002. (DOCX)

S1 Checklist STROBE statement—Checklist of items that should be included in reports of cohort studies. (DOC)
