# Supplementary material for: Frailty before and during austerity: A time series analysis of the English Longitudinal Study of Ageing 2002–2018
Source: PLoS One. 2024 Feb 7;19(2):e0296014. doi: 10.1371/journal.pone.0296014 (PMC10849239; doi:10.1371/journal.pone.0296014)
Supplement: S1 Table — (DOCX) [file pone.0296014.s001.docx]

S1: Elements comprising the frailty index score and their seven domains

| **Variable name** | **Domain (taken from elsa questionnaire)** | **Description** |
| --- | --- | --- |
| hemobwa | mobility | Difficulty walking 100m |
| hemobsi | mobility | Difficulty sitting 2 hrs |
| hemobch | mobility | difficulty getting up from chair after sitting long periods |
| hemobcs | mobility | Difficulty climbing several flights of stairs without resting |
| hemobcl | mobility | Difficulty climbing one flight of stairs without resting |
| hemobst | mobility | Difficulty stooping, kneeling or crouching |
| hemobre | mobility | Difficulty reaching or extending arms above shoulder level |
| hemobpu | mobility | Difficulty pulling or pushing large objects |
| hemobli | mobility | Difficulty lifting or carrying weights over 10 pounds (4.54kg) |
| hemobpi | mobility | Difficulty picking up a 5p coin from a table |
| headldr | activities of daily life | Difficulty dressing, including putting on shoes and socks |
| headlwa | activities of daily life | difficulty walking across a room |
| headlba | activities of daily life | Difficulty bathing or showering |
| headlea | activities of daily life | Difficulty eating, such as cutting up food |
| headlbe | activities of daily life | Difficulty getting in and out of bed |
| headlwc | activities of daily life | Difficulty using the toilet including getting up or down |
| headlma | activities of daily life | Difficulty using map to figure out how to get around a strange place |
| headlpr | activities of daily life | Difficulty preparing a hot meal |
| headlsh | activities of daily life | Difficulty shopping for groceries |
| headlph | activities of daily life | Difficulty making telephone calls |
| headlme | activities of daily life | Difficulty taking medications |
| headlhg | activities of daily life | Difficulty doing work around the house or garden |
| headlmo | activities of daily life | Difficulty managing money, eg paying bills, keeping track of expenses |
| hedimbp | Cardiovascular | High bp dx |
| hediman | Cardiovascular | Angina dx |
| hedimmi | Cardiovascular | Heart attack |
| hedimhf | Cardiovascular | Congestive heart failure |
| hedimar | Cardiovascular | Abnormal heart rhythm |
| hedimdi | Cardiovascular | Diabetes or high blood sugar |
| hedimst | Cardiovascular | Stroke dx |
| hediblu | Chronic | Lung disease dx |
| hedibas | Chronic | Asthma dx |
| hedibar | Chronic | Arthritis dx |
| hedibos | Chronic | Osteoporosis |
| hedibca | Chronic | Cancer dx |
| hedibpd | Chronic | Parkinson's dx |
| hedibps | Chronic | Psychiatric condition |
| hedibad | Chronic | Alzheimer's dx |
| hedibde | Chronic | Dementia dx |
| psceda | Psych | Whether felt depressed much of the time during the past week |
| pscedb | Psych | Whether felt everything they did during the past week was an effort |
| pscedc | Psych | Whether felt their sleep was restless during the past week |
| pscedd | Psych | Whether was happy much of the time during the past week /R |
| pscede | Psych | Whether felt lonely much of the time during the past week |
| pscedf | Psych | Whether enjoyed life much of the time during the past week /R |
| pscedg | Psych | Whether felt sad much of the time duing the past week |
| pscedh | Psych | Whether could not get going much of the time during the past week |
| hehelf | General | Self-reported general health |
| heeye | General | Self-reported eyesight (while using lenses if appropriate) |
| hehear | General | Self-reported hearing (while using hearing aid if appropriate) |
| hefla | General | Whether fallen down since last interview |
| hefrac | General | Whether has fractured hip |
| heji | General | Whether had joint replacement |
| mmpain | General | Timed walk: whether had pain whilst walking |
| cfdatd | Memory | Whether correct day of month given |
| cfdatm | Memory | Whether correct month given |
| cfdaty | Memory | Whether correct year given |
| cfday | Memory | Whether correct day given |
| cfmem | Memory | Whether prompt given for prospective memory test (remembering to write initials) |
| cflisenq | Memory | Refers to cflisen |
| cfaniq | Memory | Refers to cfani |
| cflisdq | Memory | Refers to cflisd |
|  |  |  |
| **For reference** |  |  |
| cflisen | Number of words recalled immediately | cflisenq: 1 if cflisen = 1, 0.66 if cflisen = 2, 0.33 if cflisen = 3, 0 if cflisen = 4 |
| cfani | Number of animals mentioned | cfaniq: 1 if cfani = 1, 0.75 if cfani = 2, 0.5 if cfani = 3, 0.25 if cfani = 4, 0 if cfani = 5 |
| cflisd | Number of words recalled after delay | cflisdq: 1 if cflisd = 1, 0.75 if cflisd = 2, 0.5 if cflisd = 3, 0.25 if cflisd = 4, 0 if cflisd = 5 |
