# Supplementary material for: Frailty before and during austerity: A time series analysis of the English Longitudinal Study of Ageing 2002–2018
Source: PLoS One. 2024 Feb 7;19(2):e0296014. doi: 10.1371/journal.pone.0296014 (PMC10849239; doi:10.1371/journal.pone.0296014)
Supplement: S2 Table — (DOCX) [file pone.0296014.s002.docx]

S3: AIC values for the different model interruption points in the interrupted time series analysis

| Interruption year | 2010 | 2012 | 2014 |
| --- | --- | --- | --- |
| AIC | -162205 | -162452 | -162369 |
