# Supplementary material for: Frailty before and during austerity: A time series analysis of the English Longitudinal Study of Ageing 2002–2018
Source: PLoS One. 2024 Feb 7;19(2):e0296014. doi: 10.1371/journal.pone.0296014 (PMC10849239; doi:10.1371/journal.pone.0296014)
Supplement: S3 Table — Interruption point is 2010. (DOCX) [file pone.0296014.s003.docx]

S4: Interrupted time series model output predicting the square root of the mean frailty index score. Interruption point is 2010.

| ***Predictors*** | ***Estimates (95% confidence interval)*** | ***P*** |
| --- | --- | --- |
| (Intercept) | 0·284 (0·279 to 0·289) | **<0·001** |
| Sex (binary) | .. | .. |
| Men | Reference | .. |
| Women | 0·024 (0·020 to 0·028) | **<0·001** |
| Age in 2002 (years) | .. | .. |
| <55 | Reference | .. |
| 55–59 | 0·028 (0·021 to 0·035) | **<0·001** |
| 60–64 | 0·041 (0·033 to 0·048) | **<0·001** |
| 65–69 | 0·053 (0·046 to 0·061) | **<0·001** |
| 70–74 | 0·077 (0·068 to 0·086) | **<0·001** |
| 75–79 | 0·095 (0·085 to 0·105) | **<0·001** |
| 80–84 | 0·128 (0·115 to 0·140) | **<0·001** |
| ≥85 | 0·161 (0·141 to 0·180) | **<0·001** |
| Wave (linear 1–9) | .. | .. |
| 1 = 2002-03, 9 = 2018–19 | -0·000 (-0·001 to 0·001) | 0·709 |
| Wealth tertile (categorical) | .. | .. |
| Richest | Reference | .. |
| Middle | 0·014 (0·011 to 0·017) | **<0·001** |
| Poorest | 0·045 (0·041 to 0·049) | **<0·001** |
| Period (binary 0,1) | .. | .. |
| 0 = waves 1–5 | Reference | .. |
| 1 = waves 6–9 | -0·021 (-0·025 to -0·018) | **<0·001** |
| **Interactions** | .. | .. |
| Sex*Wave | .. | .. |
| Men*wave | Reference | .. |
| Women*wave | -0·000 (-0·001 to -0·000) | **0·033** |
| Age in 2002*wave | .. | .. |
| 50–54*wave | Reference | .. |
| 55–59*wave | 0·002 (0·002 to 0·003) | **<0·001** |
| 60–64*wave | 0·005 (0·005 to 0·006) | **<0·001** |
| 65–69*wave | 0·009 (0·008 to 0·010) | **<0·001** |
| 70–74*wave | 0·012 (0·012 to 0·013) | **<0·001** |
| 75–79*wave | 0·016 (0·015 to 0·018) | **<0·001** |
| 80–84*wave | 0·019 (0·017 to 0·020) | **<0·001** |
| ≥85*wave | 0·025 (0·021 to 0·029) | **<0·001** |
| Wave * Period | 0·005 (0·004 to 0·006) | **<0·001** |
| Age in 2002*Wealth | .. | .. |
| <55*richest | Reference | .. |
| 55–59*middle | -0·002 (-0·007 to 0·003) | 0·483 |
| 60–64*middle | -0·001 (-0·006 to 0·005) | 0·811 |
| 65–69*middle | -0·005 (-0·011 to 0·000) | 0·069 |
| 70–74*middle | -0·004 (-0·011 to 0·003) | 0·314 |
| 75–79*middle | 0·001 (-0·008 to 0·009) | 0·862 |
| 80–84*middle | -0·004 (-0·015 to 0·007) | 0·479 |
| ≥85*middle | 0·003 (-0·018 to 0·023) | 0·795 |
| 55–59*poorest | -0·014 (-0·021 to -0·007) | **<0·001** |
| 60–64*poorest | -0·011 (-0·019 to -0·004) | **0·004** |
| 65–69*poorest | -0·014 (-0·022 to -0·006) | **<0·001** |
| 70–74*poorest | -0·011 (-0·020 to -0·002) | **0·020** |
| 75–79*poorest | -0·011 (-0·022 to 0·000) | 0·061 |
| 80–84*poorest | -0·019 (-0·034 to -0·005) | **0·007** |
| ≥85*poorest | -0·020 (-0·043 to 0·003) | 0·094 |
| **Random Effects** | .. | .. |
| σ^2^ | 0·004 | .. |
| τ_00_ _ij_ | 0·012 | .. |
| ICC | 0·763 | .. |
| N _j_ | 16410 | .. |
| Observations | 74190 | .. |
| Marginal R^2^ / Conditional R^2^ | 0·179 / 0·805 | .. |
