# Supplementary material for: Frailty before and during austerity: A time series analysis of the English Longitudinal Study of Ageing 2002–2018
Source: PLoS One. 2024 Feb 7;19(2):e0296014. doi: 10.1371/journal.pone.0296014 (PMC10849239; doi:10.1371/journal.pone.0296014)
Supplement: S4 Table — Interruption point is 2014. (DOCX) [file pone.0296014.s004.docx]

S5: Interrupted time series model output predicting the square root of the mean frailty index score. Interruption point is 2014.

| ***Predictors*** | ***Estimates (95% confidence interval)*** | ***P*** |
| --- | --- | --- |
| (Intercept) | 0.284 (0.270 to 0.289) | **<0.001** |
| Sex (binary) | .. | .. |
| Men | Reference | .. |
| Women | 0.024 (0.020 to 0.028) | **<0.001** |
| Age in 2002 (years) | .. | .. |
| <55 | Reference | .. |
| 55–59 | 0.027 (0.020 to 0.034) | **<0.001** |
| 60–64 | 0.040 (0.032 to 0.047) | **<0.001** |
| 65–69 | 0.052 (0.045 to 0.060) | **<0.001** |
| 70–74 | 0.076 (0.067 to 0.084) | **<0.001** |
| 75–79 | 0.094 (0.083 to 0.104) | **<0.001** |
| 80–84 | 0.126 (0.113 to 0.138) | **<0.001** |
| ≥85 | 0.159 (0.140 to 0.179) | **<0.001** |
| Wave (linear 1–9) | .. | .. |
| 1 = 2002-03, 9 = 2018–19 | 0.000 (-0.000 to 0.001) | 0.558 |
| Wealth tertile (categorical) | .. | .. |
| Richest | Reference | .. |
| Middle | 0.014 (0.011 to 0.017) | **<0.001** |
| Poorest | 0.045 (0.041 to 0.049) | **<0.001** |
| Period (binary 0,1) | .. | .. |
| 0 = waves 1–5 | Reference | .. |
| 1 = waves 6–9 | -0.030 (-0.038 to -0.021) | **<0.001** |
| **Interactions** | .. | .. |
| Sex*Wave | .. | .. |
| Men*wave | Reference | .. |
| Women*wave | -0.000 (-0.001 to -0.000) | **0.028** |
| Age in 2002*wave | .. | .. |
| 50–54*wave | Reference | .. |
| 55–59*wave | 0.003 (0.002 to 0.003) | **<0.001** |
| 60–64*wave | 0.006 (0.005 to 0.006) | **<0.001** |
| 65–69*wave | 0.009 (0.008 to 0.010) | **<0.001** |
| 70–74*wave | 0.013 (0.012 to 0.014) | **<0.001** |
| 75–79*wave | 0.017 (0.016 to 0.018) | **<0.001** |
| 80–84*wave | 0.019 (0.017 to 0.021) | **<0.001** |
| ≥85*wave | 0.026 (0.022 to 0.030) | **<0.001** |
| Wave * Period | 0.006 (0.005 to 0.007) | **<0.001** |
| Age in 2002*Wealth | .. | .. |
| <55*richest | Reference | .. |
| 55–59*middle | -0.002 (-0.007 to 0.003) | 0.468 |
| 60–64*middle | -0.001 (-0.006 to 0.005) | 0.842 |
| 65–69*middle | -0.005 (-0.011 to 0.000) | 0.066 |
| 70–74*middle | -0.003 (-0.010 to 0.003) | 0.325 |
| 75–79*middle | 0.001 (-0.008 to 0.009) | 0.860 |
| 80–84*middle | -0.004 (-0.015 to 0.007) | 0.479 |
| ≥85*middle | 0.003 (-0.018 to 0.023) | 0.797 |
| 55–59*poorest | -0.014 (-0.021 to -0.007) | **<0.001** |
| 60–64*poorest | -0.011 (-0.019 to 0.023) | **0.004** |
| 65–69*poorest | -0.014 (-0.022 to -0.006) | **<0.001** |
| 70–74*poorest | -0.011 (-0.020 to -0.002) | **0.022** |
| 75–79*poorest | -0.011 (-0.022 to 0.001) | 0.065 |
| 80–84*poorest | -0.019 (-0.033 to -0.005) | **0.009** |
| ≥85*poorest | -0.019 (-0.042 to 0.004) | 0.100 |
| **Random Effects** | .. | .. |
| σ^2^ | 0·004 | .. |
| τ_00_ _ij_ | 0·012 | .. |
| ICC | 0·763 | .. |
| N _j_ | 16410 | .. |
| Observations | 74190 | .. |
| Marginal R^2^ / Conditional R^2^ | 0·180 / 0·806 | .. |
