# Supplementary material for: Frailty before and during austerity: A time series analysis of the English Longitudinal Study of Ageing 2002–2018
Source: PLoS One. 2024 Feb 7;19(2):e0296014. doi: 10.1371/journal.pone.0296014 (PMC10849239; doi:10.1371/journal.pone.0296014)
Supplement: S5 Table — Modelled for 2002–2010 and 2012–2018 separately. (DOCX) [file pone.0296014.s005.docx]

S9: Accelerated time series model outputs predicting the square root of the mean frailty index score. Modelled for 2002-2010 and 2012-2018 separately.

| ***Predictors*** | ***Estimates (95% confidence interval)***  *Model 2002-2010* | ***P*** | ***Estimates (95% confidence interval)***  *Model 2012-2018* | ***P*** |
| --- | --- | --- | --- | --- |
| (Intercept) | 0·278 (0·272 to 0·284) | **<0·001** | 0·245 (0·234 to 0·257) | **<0·001** |
| Sex (binary) | .. | .. | .. | .. |
| Men | Reference | .. | Reference | .. |
| Women | 0·023 (0·019 to 0·027) | **<0·001** | 0·023 (0·018 to 0·028) | **<0·001** |
| Age in 2002 (years) | .. | .. | Age in 2012 | .. |
| 50-54 | Reference | .. | Reference | .. |
| 55-59 | 0.024 (0.015 to 0.032) | **<0·001** | 0.029 (0.015 to 0.043) | **<0·001** |
| 60–64 | 0.033 (0.024 to 0.042) | **<0·001** | 0.031 (0.017 to 0.044) | **<0·001** |
| 65–69 | 0·051 (0·042 to 0·061) | **<0·001** | 0·051 (0·037 to 0·064) | **<0·001** |
| 70–74 | 0·076 (0·066 to 0·086) | **<0·001** | 0·075 (0·061 to 0·089) | **<0·001** |
| 75–79 | 0·095 (0·084 to 0·106) | **<0·001** | 0·100 (0·086 to 0·114) | **<0·001** |
| 80–84 | 0·131 (0·117 to 0·144) | **<0·001** | 0·133 (0·116 to 0·151) | **<0·001** |
| ≥85 | 0·177 (0·157 to 0·197) | **<0·001** | 0·174 (0·155 to 0·194) | **<0·001** |
| Wave (linear 1–5) | .. | .. | Wave (linear 1–4) | .. |
| 1 = 2002-03, 5 = 2010–11 | .. | .. | 1= 2012-13, 4= 2018–19 | .. |
|  | 0·003 (0·001 to 0·004) | **<0·001** | 0·006 (0·004 to 0·009) | **<0·001** |
| Wealth tertile (categorical) | .. | .. | .. | .. |
| Richest | Reference | .. | Reference | .. |
| Middle | 0·019 (0·014 to 0·024) | **<0·001** | 0·016 (0·008 to 0·025) | **<0·001** |
| Poorest | 0·067 (0·060 to 0·073) | **<0·001** | 0·064 (0·053 to 0·075) | **<0·001** |
| **Interactions** | .. | .. | **..** | .. |
| Age in 2002*wave | .. | .. | Age in 2012*wave | .. |
| 50-54*wave | Reference | .. | Reference | .. |
| 55-59*wave | 0.002 (0.000 to 0.003) | **0.031** | 0.001 (-0.002 to 0.003) | 0.613 |
| 60–64*wave | 0.005 (0.003 to 0.006) | **<0.001** | 0.003 (0.001 to 0.006) | **0.009** |
| 65–69*wave | 0·008 (0·006 to 0·009) | **<0·001** | 0·006 (0·003 to 0·009) | **<0·001** |
| 70–74*wave | 0·012 (0·011 to 0·014) | **<0·001** | 0·010 (0·007 to 0·013) | **<0·001** |
| 75–79*wave | 0·015 (0·013 to 0·017) | **<0·001** | 0·014 (0·011 to 0·017) | **<0·001** |
| 80–84*wave | 0·017 (0·015 to 0·020) | **<0·001** | 0·017 (0·014 to 0·021) | **<0·001** |
| ≥85*wave | 0·016 (0·011 to 0·021) | **<0·001** | 0·028 (0·024 to 0·033) | **<0·001** |
| Age in 2002*Wealth | .. | .. | *Age in 2012 | .. |
| 50-54*richest | Reference | .. | Reference | .. |
| 55-59*middle | -0.001 (-0.008 to 0.007) | 0.836 | -0.001 (-0.012 to 0.011) | 0.919 |
| 60–64*middle | 0.005 (-0.003 to 0.013) | 0.262 | -0.001 (-0.012 to 0.010) | 0.815 |
| 65–69*middle | -0·004 (-0·012 to 0·004) | 0·322 | -0·001 (-0·012 to 0·010) | 0·873 |
| 70–74*middle | -0·008 (-0·017 to 0·001) | 0·067 | -0·003 (-0·014 to 0·009) | 0·645 |
| 75–79*middle | -0·003 (-0·013 to 0·007) | 0·574 | -0·005 (-0·017 to 0·007) | 0·414 |
| 80–84*middle | -0·010 (-0·022 to 0·002) | 0·110 | -0·002 (-0·018 to 0·013) | 0·748 |
| ≥85*middle | -0·005 (-0·025 to 0·016) | 0·648 | -0·002 (-0·020 to 0·015) | 0·787 |
| 55-59*poorest | -0.021 (-0.030 to -0.011) | **<0.001** | -0.004 (-0.018 to 0.013) | 0.547 |
| 60-64*poorest | -0.011 (-0.021 to -0.001) | **0.033** | -0.020 (-0.033 to -0.006) | **0.004** |
| 65–69*poorest | -0·026 (-0·036 to -0·015) | **<0·001** | -0·019 (-0·032 to -0·005) | **0·007** |
| 70–74*poorest | -0·027 (-0·039 to -0·016) | **<0·001** | -0·026 (-0·041 to -0·011) | **<0·001** |
| 75–79*poorest | -0·023 (-0·036 to -0·009) | **0·001** | -0·025 (-0·040 to -0·010) | **0·001** |
| 80–84*poorest | -0·037 (-0·052 to -0·022) | **<0·001** | -0·025 (-0·044 to -0·006) | **0·011** |
| ≥85*poorest | -0·043 (-0·066 to -0·019) | **<0·001** | -0·039 (-0·061 to -0·018) | **<0·001** |
| **Random Effects** | .. | .. | .. | .. |
| σ^2^ | 0·003 | .. | 0.003 | .. |
| τ_00_ _ij_ | 0·011 | .. | 0.013 | .. |
| ICC | 0·765 | .. | 0.808 | .. |
| N _j_ | 14403 | .. | 10052 | .. |
| Observations | 44496 | .. | 29694 | .. |
| Marginal R^2^ / Conditional R^2^ | 0·175 / 0·806 | .. | 0.177 / 0.842 | .. |
