# Supplementary material for: Frailty before and during austerity: A time series analysis of the English Longitudinal Study of Ageing 2002–2018
Source: PLoS One. 2024 Feb 7;19(2):e0296014. doi: 10.1371/journal.pone.0296014 (PMC10849239; doi:10.1371/journal.pone.0296014)
Supplement: S2 File — (DOCX) [file pone.0296014.s007.docx]

S8: Accelerated times series model specification

For waves 1-5, let

$f_{ij}$= frailty index value at time (i=1…5) for individual j

$T_{ij}$= Time since 2002 at wave i for individual j

$A_{1j}$= Age group based on age at 2002 (Mid-points of five-year age bands)

$S_{j}$= Sex of individual j

$W_{ijk}$= Wealth tertile at wave i for individual j born in decade k (where 1 = richest third, 3 = poorest third)

For waves 6-9, let

$f_{ij}$= frailty index value at time (i=1…4) for individual j

$T_{ij}$= Time since 2012 at wave i for individual j

$A_{1j}$= Age group based on age at 2012 (Mid-points of five-year age bands)

$$S_{j} as for waves 1-5 \mathrm{model}$$

$$W_{ijk} as for waves 1-5 model$$

$$sqrt\left( f_{ij} \right)=B_{0j}+B_{1}\left( S_{ij} \right)+B_{2}\left( A_{1j} \right)+B_{3}\left( T_{ij} \right)+B_{4}\left( W_{ijk} \right)+B_{5}\left( A_{1j}*T_{ij} \right)+B_{6}\left( A_{1j}*W_{ijk} \right){+ e}_{ij}$$

Where:

$$B_{0j}=B_{0}+U_{j}$$

$$U_{j}\sim N\left( 0,\sigma_{u}^{2} \right)$$

$$e_{ij}\sim N\left( 0,\sigma_{e}^{2} \right)$$
