# Supplementary material for: Frailty before and during austerity: A time series analysis of the English Longitudinal Study of Ageing 2002–2018
Source: PLoS One. 2024 Feb 7;19(2):e0296014. doi: 10.1371/journal.pone.0296014 (PMC10849239; doi:10.1371/journal.pone.0296014)

S7: Modelled mean frailty over time for different age groups, stratified by wealth tertile and sex using an interrupted time series approach with interruption in 2014


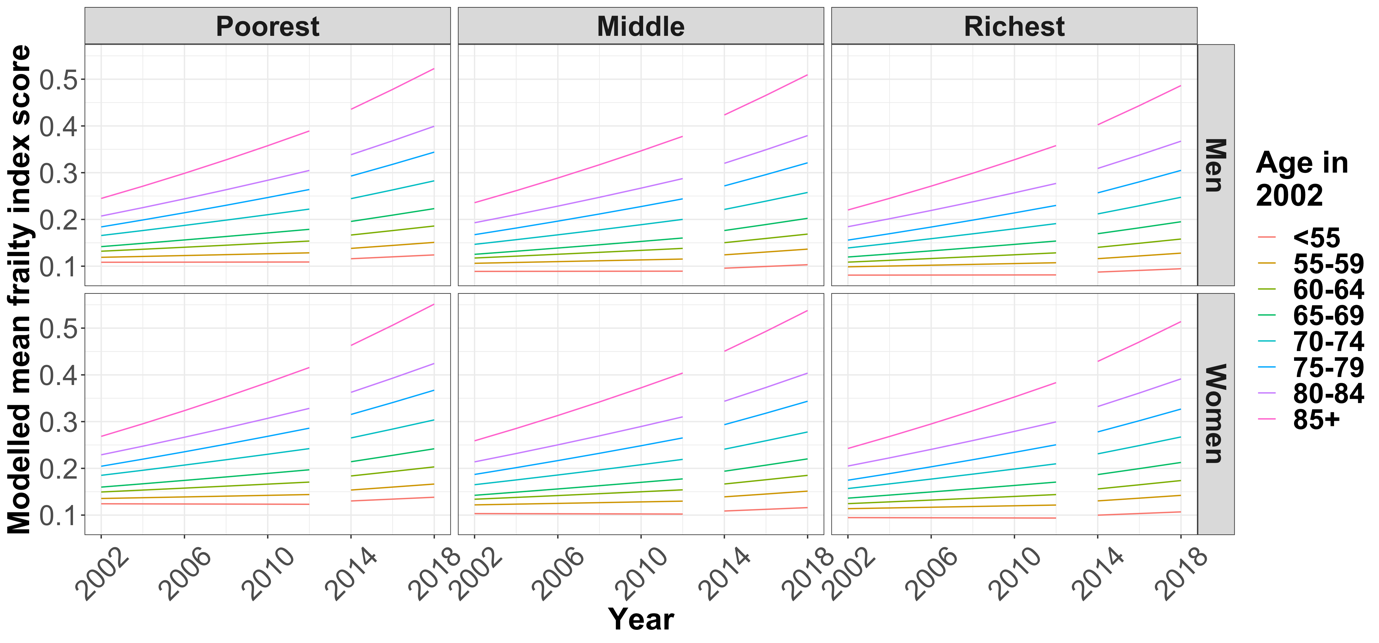

Supplement: S2 Fig — (DOCX) [file pone.0296014.s009.docx]
