# Supplementary material for: Frailty before and during austerity: A time series analysis of the English Longitudinal Study of Ageing 2002–2018
Source: PLoS One. 2024 Feb 7;19(2):e0296014. doi: 10.1371/journal.pone.0296014 (PMC10849239; doi:10.1371/journal.pone.0296014)

S10. Mean proportion of deficits for each domain, at each wave of data collection, stratified by age in 2002


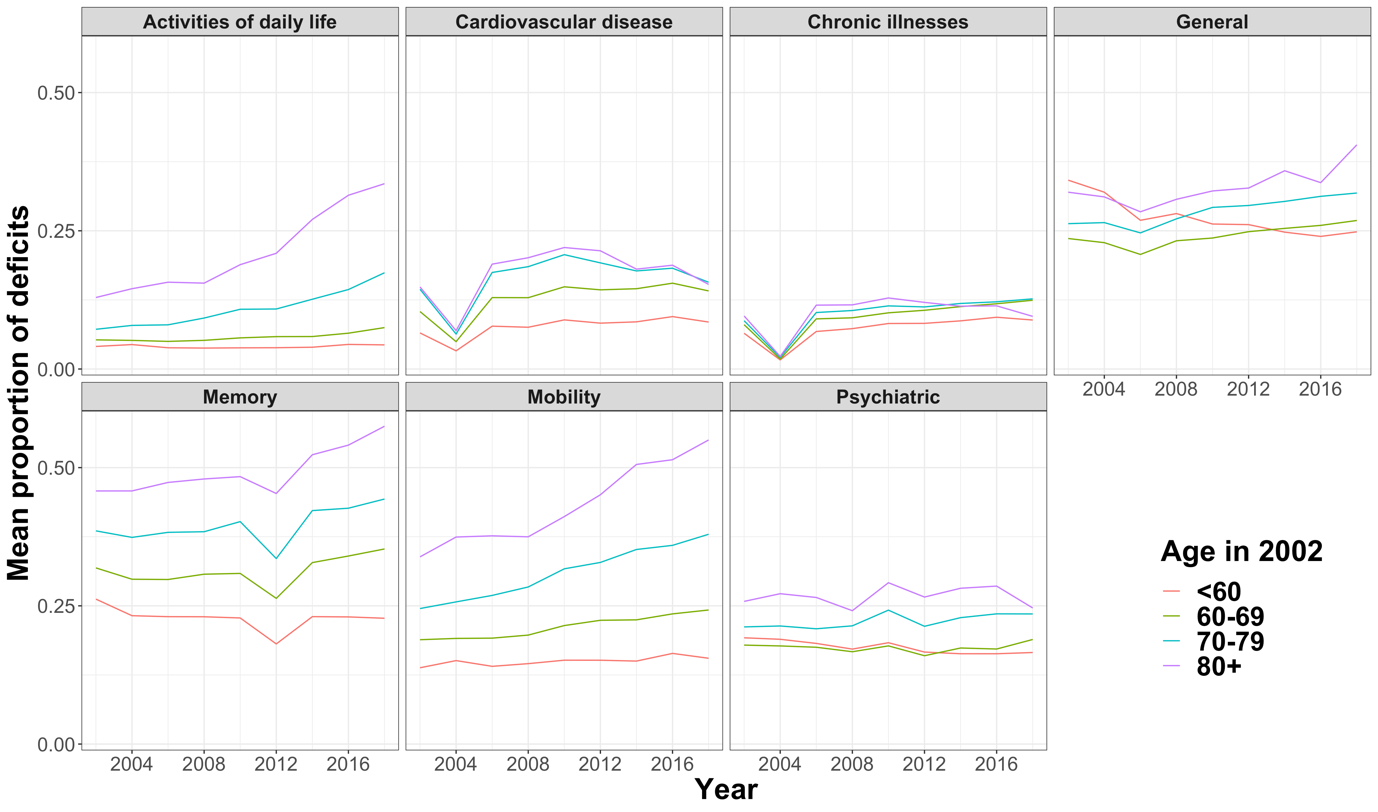

Supplement: S3 Fig — (DOCX) [file pone.0296014.s010.docx]
